# Potential of Selected C-X-C Motif Chemokines as Biomarkers in Colorectal Cancer Diagnosis

**DOI:** 10.3390/ijms26178715

**Published:** 2025-09-07

**Authors:** Adrianna Romanowicz, Marta Łukaszewicz-Zając, Barbara Mroczko

**Affiliations:** 1 Department of Biochemical Diagnostics, Medical University of Bialystok, 15-269 Bialystok, Poland; adrianna.romanowicz@sd.umb.edu.pl (A.R.); mroczko@umb.edu.pl (B.M.); 2 Department of Neurodegeneration Diagnostics, Medical University of Bialystok, 15-269 Bialystok, Poland

**Keywords:** biomarker, chemokines, colorectal cancer

## Abstract

Colorectal cancer (CRC) is a leading cause of cancer-related morbidity and mortality worldwide, mainly due to late diagnosis and high metastatic potential. Effective management requires accurate diagnostic, prognostic, and therapeutic strategies, with growing focus on molecular biomarkers. Chemokines, which are small, secreted proteins regulating immune cell migration and tissue homeostasis, shape the tumor microenvironment by promoting tumor growth, angiogenesis, immune evasion, and metastasis. In CRC, the expression of altered chemokine–receptor profiles correlates with progression and clinical outcomes. Chemokines are classified by the presence or absence of the ELR motif, which differentiates CXC subgroups. Selection focused on those consistently altered in CRC tissues or serum and involved in key oncogenic processes. CXCL1 and its receptor CXCR2 are overexpressed and linked to tumor progression, highlighting their diagnostic and therapeutic potential. CXCL8 is elevated in tissues and serum, correlating with metastasis and poor survival. The CXCL12/CXCR4/CXCR7 axis drives metastasis. CXCL13 promotes immune evasion via CXCR5, while CXCL14 is downregulated, suggesting a protective role. Moreover, CXCL16 associates with worse outcomes, whereas CXCR6 may enhance immunotherapy response. Overall, chemokines and receptors are promising blood biomarkers and therapeutic targets in CRC. Further validation is needed using large prospective studies, standardized assays, and multi-marker approaches to establish their potential as non-invasive CRC biomarkers.

## 1. Colorectal Cancer—Epidemiology, Mortality and Risk Factors

Colorectal cancer (CRC) is one of the most diagnosed malignancies worldwide, currently ranking third in terms of global incidence and second in cancer-related mortality [1,2]. Data from the current report of the International Agency for Research on Cancer indicate that approximately 1.9 million new cases of CRC were identified in 2022, accompanied by around 904,000 deaths, resulting in a mortality rate of nearly 20% [2]. Although overall CRC incidence trends have stabilized in the general population, there is accumulating evidence of a worrying increase in early-onset CRC diagnosed before age 50, suggesting a demographic shift towards younger individuals [3]. The epidemiologic data obtained from North America and West Europe have reported annual increases of 2–3% in individuals under 50 since 2022, while some Asian and African regions show slower but emerging trends. From 1990 to 2021, early-onset CRC cases nearly doubled, rising from about 107,000 to 212,000, with the age-standardized incidence rate increasing from 4.58 to 5.15 per 100,000, further highlighting the growing clinical urgency for early detection strategies [1,2,3]. A multistep carcinogenic process is known to be responsible for most CRCs, which arise from precancerous lesions, particularly adenomatous polyps. CRC can be classified as sporadic, hereditary and familial types depending on the origin of the underlying mutations [4]. The pathogenesis of CRC is multifactorial, involving a complex interplay between genetic predisposition and environmental exposures. Sporadic cases account for over 70% of all CRC diagnoses and are primarily associated with modifiable lifestyle factors. It is estimated that around 75% of CRC cases arise from the interplay between environmental factors and genetic predisposition. Besides age and genetics, several lifestyle risk factors have been identified, including a diet low in fruits and vegetables, excessive consumption of red and processed meat, high saturated fat and alcohol intake, tobacco use, and obesity [5,6]. Although inherited forms of CRC account for a relatively small proportion of cases, they are clinically significant. Mutations in key genes, such as oncogenes, tumor suppressor genes, and genes involved in DNA repair pathways, can initiate and drive colorectal tumorigenesis [5,6]. It is estimated that hereditary factors contribute to approximately 20% of CRC cases, classified as familial CRC [7,8].

Worldwide, nearly 60% of CRC cases are diagnosed in high-income countries, with Europe reporting some of the highest incidence and mortality rates associated with the disease [5]. There is an urgent need for reliable diagnostic tools capable of detecting the disease at an early stage, given the significant burden of CRC in terms of both mortality and morbidity. Prognosis is closely linked to the stage at which CRC is recognized. The overall five-year relative survival rate is estimated to be 50–60%, but this figure rises to approximately 80% if the disease is detected at an early stage. Conversely, once CRC has progressed to advanced stages, the five-year survival rate falls below 15% [9,10]. Unfortunately, CRC is often diagnosed only after the onset of clinical symptoms, which typically manifest at more advanced stages of tumor development.

### An Overview of Current Diagnostic and Screening Strategies for Colorectal Cancer

**Figure 1:**
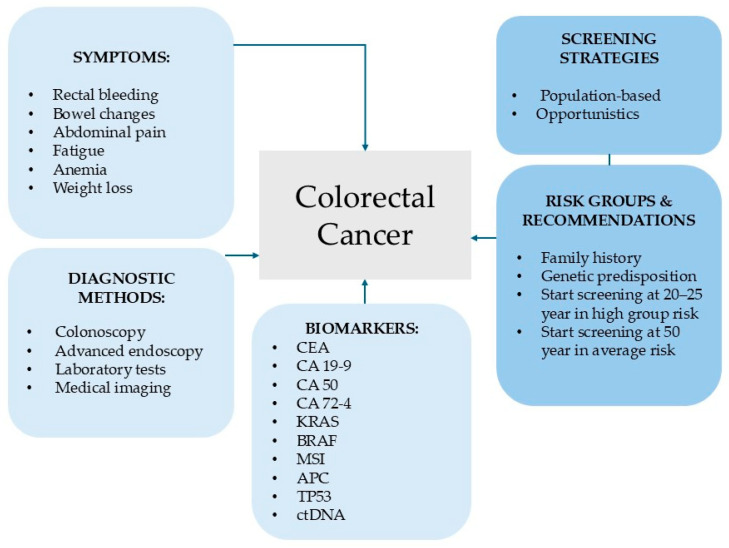
Current diagnostic and screening strategies for colorectal cancer.

Patients with CRC may have symptoms such as rectal bleeding, bowel irregularities, abdominal discomfort, chronic fatigue, anemia, or unintentional weight loss. Interestingly, most CRC cases occur in men and women over the age of 50 who have no clinical symptoms and no personal or family history of this disease. The diagnostic process for CRC is multifaceted, involving both laboratory analysis and medical imaging. Among tumor markers, carcinoembryonic antigen (CEA) is the most widely studied, but its low sensitivity in early-stage cancer limits its usefulness in screening protocols [Figure 1]. Alternative biomarkers, such as cancer antigen 19-9 (CA 19-9), cancer antigen 50 (CA 50), and cancer antigen 72.4 (CA 72.4), have also been evaluated. Other relevant biomarkers include Kirsten rat sarcoma viral oncogene (KRAS), Raf murine sarcoma viral oncogene homolog (BRAF) mutations, microsatellite instability (MSI), adenomatous polyposis coli (APC), tumor protein p53 (TP53) and circulating tumor DNA (ctDNA), which are increasingly used in clinical practice for diagnostic, prognostic, and predictive purposes [11,12,13]. Despite significant efforts over the past decade to discover effective early detection markers, there is still no universally accepted biomarker for the identification of CRC in its initial stages. This highlights the urgent need to develop noninvasive and reliable diagnostic tools, such as blood-based markers, to support early diagnosis and improve patient prognosis.

At present, CRC screening generally be divided into population-based and opportunistic approaches. Population-based programs, typically implemented by public health systems, target early or asymptomatic cases, but are resource intensive and often suffer from low participation rates. Opportunistic screening, conducted during regular medical visits, is more cost-effective and tends to achieve better compliance. However, its reach is limited because it often excludes individuals without symptoms, reducing its overall impact on disease incidence [14]. Extensive research supports lowering the age of initial screening for individuals at increased risk of CRC, including those with a family history of CRC, microsatellite instability, deficient mismatch repair deficiency, or inherited genetic mutations. For example, individuals with Lynch syndrome should begin screening between the ages of 20 and 25, or 2 to 10 years earlier than the youngest affected family member. In cases of familial adenomatous polyposis, colonoscopy may be recommended as early as 10 to 18 years age [15]. Current early screening methods include risk assessment questionnaires, the Asia-Pacific Colorectal Screening Score (APCS), fecal immunochemical testing (FIT), multi-target fecal DNA analysis, circulating tumor cell detection, virtual colonoscopy, and conventional colonoscopy [16]. The APCS uses basic clinical factors such as age, gender, family history, and smoking status, and is a practical tool for use by primary care providers [17]. A large clinical study involving nearly 10,000 participants found that FIT had a sensitivity of 73.8% and a specificity of 94.9% for the detection of CRC. Combining FIT with questionnaire-based risk stratification or APCS scoring is an efficient and cost-effective strategy for large-scale screening programs. Despite these advances, colonoscopy remains the most accurate and reliable method for diagnosing CRC [16].

## 2. Inflammation and Chemokines in Cancer Progression

Chemokines are a diverse family of small cytokines characterized by low molecular weight and structural similarity. Based on the arrangement of the conserved N-terminal cysteine residues, they have been classified into four major subfamilies: CXC, CC, CX3C, and XC [18,19]. These proteins exert their biological effects through interactions with specific G protein-coupled receptors (GPCRs) that span the cell membrane seven times [20]. Each receptor can respond to several chemokines, and individual chemokines can activate multiple types of receptors, contributing to a complex and multifaceted signaling network [21].

Functionally, chemokines play a central role in guiding the migration (chemotaxis) and positioning of a variety of immune cells such as lymphocytes, neutrophils, eosinophils, and fibroblasts [22]. Chemokines from the CXC family can be categorized based on the presence or absence of the ELR (Glu-Leu-Arg) motif preceding the first cysteine residue in their structure. ELR+ chemokines, such as C-X-C motif chemokine ligand 1 (CXCL1) and 8 (CXCL8), contain this tripeptide sequence and are known to interact primarily with C-X-C chemokine receptor 1 (CXCR1) and receptor 2 (CXCR2), contributing to the recruitment of neutrophils and promoting angiogenesis [23,24]. In contrast, ELR- chemokines such as C-X-C motif chemokine ligand 12 (CXCL12), ligand 13 (CXCL13), ligand 14 (CXCL14), and ligand 16 (CXCL16) lack the ELR motif. CXCL12 promotes angiogenesis and contributes to tumor progression. CXCL13, expressed by stromal cells and macrophages, is a key chemoattractant for B cells and has been implicated in cancer development [25]. CXCL14, a highly conserved chemokine, regulates immune cell infiltration and dendritic cell maturation, with its role in tumor growth varying by cellular origin—promoting it when derived from fibroblasts and inhibiting it when produced by epithelial cells [26]. CXCL16, abundantly expressed in organs such as the liver, lung, and kidney, recruits natural killer cells involved in antitumor immunity [27]. In addition, chemokines have also been involved in a variety of pathological conditions, including autoimmune diseases, atherosclerosis, HIV infection, and cancer [28,29,30]. In the context of malignant tumors, chemokines and their receptors have been increasingly recognized as critical regulators of tumor biology. In CRC, they contribute to multiple stages of tumorigenesis, such as cell proliferation, angiogenesis, intravascular survival, endothelial adhesion, and extravasation into secondary sites [31,32]. They also shape the tumor microenvironment (TME) by enabling crosstalk between the tumor and the surrounding non-tumor cells, thereby modulating the activity of neutrophils and tumor-associated macrophages [33].

Chemokine receptors also play a crucial role in regulating the migration and function of monocytes and their subtypes within the tumor microenvironment of CRC [34]. They are essential for directing monocytes into the CRC microenvironment. These monocytes then differentiate into various subtypes of TAMs, including M-like (anti-tumor) and M2-like (pro-tumor) macrophages. Pro-inflammatory M1 can be activated by IFN-γ, lipopolysaccharide (LPS) or tumor necrosis factor-α (TNF-α), which subsequently activate Toll-like receptor signaling pathways as well as promotes type 1 T helper (Th1) antitumor immune response by producing cytokines such as TNF-α, IL-1α, IL-1β, IL-6, IL-12, IL-18 and IL-23. Anti-inflammatory M2 macrophages can be induced by cytokines such as IL-4, IL-10, glucocorticoid, M-CSF, transforming growth factor-β (TGF-β) and via further differentiation can be subdivided into M2a, M2b, M2c and M2d subgroups. In addition, M2 macrophages participate in Th2 immune response, including humoral immunity and anti-inflammatory, while in solid tumors such as CRC, M2 macrophages promote tumor progression and invasion by inducing angiogenesis and suppressing the host immune response. Table 1 provides a concise comparison of myeloid and lymphoid populations, including chemokine receptor usage and their roles in CRC pathogenesis [34].

Inflammatory process plays a critical role in the body’s defense mechanisms and is characterized by complex changes in molecular and cellular components. While essential for pathogen clearance and tissue repair, chronic inflammation may contribute to tumorigenesis, including CRC [35,36,37]. Numerous studies have confirmed that inflammatory responses contribute to carcinogenesis. Tumors often harbor inflammatory cells that secrete pro-inflammatory mediators, that promote tumor growth and metastasis within the TME [38]. These signaling pathways support cancer cell proliferation and migration [35].

Accumulating evidence highlights the central role of chemokines, key pro-inflammatory mediators, in shaping tumor cell behavior through autocrine and paracrine signaling. Chemokines influence processes such as cell migration, proliferation, immune evasion, and neoangiogenesis [39]. They are known to be critical in regulating metastasis [31]. Metastatic progression involves multiple steps, including tumor cell adhesion, intravasation, circulation, and colonization of distant tissues. Clinical studies show that chemokines support these steps by directing tumor cell movement, enhancing angiogenesis, and protecting cells from immune responses [40]. Originally known for guiding immune cells, chemokines also affect non-immune cells that are integral to tumor growth [41]. Tumor cells often gain the ability to express chemokine receptors and produce chemokines themselves, further driving cancer progression [42]. An important process they influence is angiogenesis, the formation of new blood vessels from existing ones. This process ensures the supply of oxygen and nutrients to the tumor and removes waste products. Some chemokines are stimulators of angiogenesis, while others are inhibitors of their functions [41]. This dual function is well documented in melanoma, which expresses chemokines such as CCL2, CCL5, CXCL1, CXCL2, CXCL3, and CXCL8 which have been implicated in tumor growth and progression [43].

Metastasis, defined as the dissemination of cancerous cells to distant organs, constitutes a pivotal and often lethal phase of cancer. The release of chemokines by target tissues has been demonstrated to attract circulating tumor cells, thereby enabling the formation of secondary tumors [44]. A chemokine axis that has been the focus of significant research is the interaction between CXCR4 and its ligand CXCL12. Inhibition of this pathway has been demonstrated to reduce metastasis in breast cancer, likely through the disruption of CXCL12/CXCR4 signaling, which impairs chemotactic and invasive responses of tumor cells and limits their spread to regional lymph nodes and lungs. This axis has also been implicated in prostate, lung, and CRC [45,46,47,48]. Several members of the CXC chemokine family and their corresponding receptors have been identified as key factors in CRC development and may serve as potential biomarkers for diagnosis, prognosis, or therapeutic targets [48,49,50]. This review aims to provide a comprehensive analysis of selected CXC chemokines, both ELR+ and ELR- (CXCL1, CXCL8, CXCL12, CXCL13, CXCL14, and CXCL16), along with their corresponding receptors, in the context of CRC. The primary objective of our review is to evaluate existing research on chemokines expression patterns, functional roles, and involvement in key oncogenic processes, to assess their potential utility as non-invasive diagnostic, prognostic, and therapeutic biomarkers.

**Table 1:** Overview of myeloid and lymphoid populations in colorectal cancer.

Lineage	Cell Type	CXC Receptor/Ligand	Role in CRC	References
Myeloid	Neutrophils (TANs)	CXCR1/2, CXCL8	CXCL8-CXCR1/2 axis promotes neutrophil recruitment, angiogenesis, proliferation, EMT, NET formation, immunosuppression and metastasis. Elevated serum IL-8 correlated with increased TAN density and poor response to immune checkpoint inhibitors.	[51]
	Macrophages (TAMs)	CXCL12, CXCL1	Recruit TAMs into the tumor via increased IL-1, IL-6 and TNF-α production. In CRCLM blockade of CXCL12 increases M1/M2 macrophage ratio, shifting towards anti-tumor phenotype. CXCL1 produced by TAMs recruits CXCR2+ MDSCs for the pre-metastatic niche to stimulate liver metastases in CRC	[52,53]
Lymphoid	T cells (CD8+/Th1)	CXCR3, CXCL9, CXCL10, CXCL11	Recruit effector T-cell into the tumor. Increased expression of CXCL9/10 linked to favorable prognosis.	[54]
	B cells (TLS)	CXCL13	Recruits B cells and promotes TLS formation. CXCL13 expression in CRC correlates with improved survival among CRC patients.	[55]

EMT—epithelial–mesenchymal transition; NET—neutrophil extracellular trap; TAN—tumor-associated neutrophil; TAM—tumor-associated macrophage; IL-1, IL-6—interleukin 1,6; TNF-α—tumor necrosis factor alpha; CRCLM—colorectal cancer liver metastasis; MDSC—myeloid-derived suppressor cell; TLS—tertiary lymphoid structure.

## 3. Functional Roles of Chemokines in Colorectal Cancer Pathogenesis

### 3.1. ELR+ Chemokines

#### 3.1.1. CXCL8, Its Receptors, and the Related Signaling Axis in Colorectal Cancer

CXCL8 binds to the receptors CXCR1 and CXCR2, which helps recruit and activate neutrophils to areas where there is inflammation or infection. It can work as a single molecule or as a pair, and these two forms can have slightly different effects depending on which receptor they bind to. CXCL8 plays a key role in the body’s response to infection—it not only attracts neutrophils, but also helps them release enzymes and reactive oxygen molecules to fight off harmful agents [56]. Both the monomeric and dimeric forms of CXCL8 interact with CXCR1 and CXCR2 but differ in their binding affinities and the signaling pathways they activate [57,58]. CXCR1 mainly responds to CXCL8, while CXCR2 also reacts to other similar chemokines, such as CXCL1 [59]. Interestingly, CRC can produce CXCL8 themselves and increase the levels of CXCR1 and CXCR2, which helps the tumor grow, spread, and form metastases [59].

A variety of studies have demonstrated that CXCL8 shows up not only in cancer cells but also in the cells lining blood vessels and in macrophages connected to tumors, including in CRC [60]. Tests using staining methods showed that their levels were clearly higher in cancer tissues than in samples from inflamed or non-malignant tissues [61]. Higher CXCL8 expression in CRC tissue have been linked to more advanced disease stages, larger tumor size, deeper tissue invasion, distant metastases, and faster cancer progression. This suggests that CXCL8 might be a useful marker for tracking how the disease develops [60,62,63,64]. The study demonstrated that high CXCL8 expression in the stromal compartment of the tumor, combined with increased numbers of CD68+ macrophages or elevated systemic neutrophil counts, was associated with poorer prognosis in CRC patients compared to healthy individuals [65]. High stromal CXCL8 expression was also associated with mutations in the CREB-binding protein (CREBBP) gene, a regulator involved in immune modulation. Furthermore, CXCR2+ immune cells were more abundant in stroma-rich primary and metastatic tumor tissues, suggesting a role for the CXCL8/CXCR2 axis in promoting an unfavorable tumor phenotype and supporting immunosuppression. Combining CXCL8/CXCR2-targeted therapies with standard chemotherapy may represent a promising therapeutic strategy in CRC [65]. In addition, researchers have demonstrated that high CXCL8 expression in CRC tissues is significantly associated with poorer overall survival (OS) in CRC patients, with pooled analyses showing over a twofold increased risk of death [66]. Recent findings indicate that CXCL8 production by cancer cells in vitro is regulated at multiple levels and is strongly influenced by the tumor’s genetic profile, particularly mutations in genes such as *BRAF* and *Phosphatase and Tensin Homolog (PTEN)* [67]. Interestingly, the loss of PTEN was significantly associated with reduced IL-8 expressions specifically in tumor-infiltrating mononuclear cells, as demonstrated by a strong inverse correlation (*p* = 0.003) [66]. In addition, Ogawa et al. reported that the loss of SMAD4 in CRCs leads to increased CXCL8 expression and secretion, which promotes neutrophil recruitment via the CXCR2 receptor [68]. Furthermore, neutrophils exposed to soluble factors derived from SMAD4-deficient CRCs exhibit upregulated CXCL8 expression, thereby amplifying the inflammatory signaling within the tumor microenvironment. Analysis of clinical samples revealed that in SMAD4-negative cancers, the number of neutrophils in the peritumor stroma was markedly higher than in SMAD4-positive cases, and the CXCL8 was strongly expressed by infiltrating neutrophils in the tumor. Neutrophils isolated directly from the tumor showed significantly higher expression of this chemokine than those from the peripheral blood.

In a study conducted by Paczek et al., authors compared the clinical significance of CXCL8 serum levels in CRC patients to the well-known tumor marker for CRC, such as CEA. Serum CXCL8 concentrations were notably elevated in CRC patients compared to control group, like the increase in CEA levels [69]. These findings were consistent with those of the other researchers, who noticed that higher serum CXCL8 levels were strongly associated with more advanced stages of CRC [70]. Furthermore, serum CXCL8 levels were markedly higher in CRC patients with distant metastases compared to those without metastases [69]. Moreover, serum CXCL8 concentration was significantly elevated in patients with CRC, especially in stages II and III, and correlated with shorter overall and relapse-free survival (RFS). These results suggest that the CXCL8/CXCR2 pathway may represent a novel therapeutic target, and serum CXCL8 levels could be a potential biomarker in CRC patients [68].

#### 3.1.2. CXCL1, Its Receptor, and Related Signaling Pathways in Colorectal Cancer

CXCL1, also called growth-regulated oncogene alpha (GROα), is a member of the CXC chemokine family and is produced by cells such as macrophages, neutrophils, and epithelial cells. It exerts its effects by binding to the G protein-coupled CXCR2 [71]. This receptor helps release myeloid cells from the bone marrow and guides them toward tumor areas with high CXCL1 levels. At these sites, the cells contribute to immune evasion by the tumor by reducing the growth, movement, and activation of effector T cells and promoting the expansion of regulatory T cells (Tregs) [72,73]. An increasing number of studies suggest that CXCL1 is involved in attracting immune cells, supporting tissue repair, and stimulating angiogenesis. This chemokine contributes to tumor development by recruiting neutrophils into the tumor microenvironment [53]. Ogata et al. demonstrated through reverse transcription polymerase chain reaction (RT-PCR) and immunohistochemical (IHC) analysis that many human CRCs produce CXCL1, and that CXCR2 mRNA is present in all tested CRC lines [74]. Similarly, Baier et al. observed that CXCL1 expression was significantly higher in cancerous colorectal tissue compared to normal samples [75]. In line with these findings, Bandpalli et al. also reported that CXCL1 was highly expressed in CRCs and was associated with increased tumor growth [76]. In addition, elevated expression of CXCL1 in CRC tissue has been strongly linked with key clinical and pathological characteristics [77]. The overproduction of CXCL1 was notably related to larger tumor size, more advanced stage, deeper invasion, and spread to lymph nodes [77]. Wang et al. proposed that CXCL1 might promote CRC metastasis through its interaction with the CXCR2 receptor [53]. 

The researchers concluded that the CXCL1/CXCR2 pathway plays a pivotal role in the development and progression of CRC in humans [74]. Consistent with the involvement of p53 mutations in tumor development, several studies have demonstrated that mutated p53 can bind directly to the promoter region of the CXCL1 gene, thereby increasing its expression in CRCs [78,79]. Based on this, researchers suggest that CXCL1 may play a significant role in CRC cases harboring p53 mutations, given its elevated expression in a substantial proportion of these tumors. Consequently, blocking the CXCL1/CXCR2 signaling axis in tumor cells could be a promising approach to limit invasion and metastasis in a wide subset of CRC patients [75]. In support of this, inhibition of the CXCL1/CXCR2 pathway has been reported to reduce the incidence of liver metastases originating from colon cancer [74,80]. These findings indicate that targeting this signaling pathway might offer a valuable therapeutic strategy in CRC treatment. The study conducted by Kong et al. was the first to demonstrate that CXCL1 suppresses CD8+ T cell proliferation, cytotoxic activity, and chemotaxis, thereby facilitating immune evasion in CRC through autophagy-mediated degradation of MHC class I molecules [81]. This may reflect that CXCL1 serve as a novel biomarker for CRC and that its inhibition could represent a promising strategy to counteract immune escape and enhance antitumor immune responses [81].

Furthermore, a recent study investigated the molecular mechanisms by which CXCL1 contributes to CRC [82]. The researchers hypothesized that the protein may influence disease progression through the NF-κB/P300 pathway. Their analysis showed markedly higher expression of CXCL1 in cancerous tissues compared to healthy ones. When the CXCL1 gene was silenced in colon cancer cells, a slowdown in their growth was observed; the opposite effect was obtained when it was overexpressed. In a mouse model, it was shown that application of the P300 inhibitor (C646) effectively inhibited tumor growth even when CXCL1 levels were increased. Additional bioinformatics analyses using the Cancer Genome Atlas (TCGA) database confirmed the clinical relevance of the results. Based on these observations, the authors concluded that CXCL1 could be an important therapeutic biomarker in CRC and a starting point for the development of new targeted therapies [82].

### 3.2. ELR- Chemokines

#### 3.2.1. CXCL12, Its Receptors, and Related Signaling Pathways in Colorectal Cancer

CXCL12, a member of the CXC chemokine family also referred to as stromal cell-derived factor-1 (SDF-1) It is a homeostatic signaling molecule that interacts with the receptors C-X-C chemokine receptor 4 (CXCR4) and C-X-C chemokine receptor 7 (CXCR7) [83]. One of its primary roles involves directing the movement of hematopoietic cells and maintaining the structural integrity of secondary lymphoid organs. CXCL12 is constantly produced in the lungs, bone marrow and liver, which is one of the main organs affected by colon cancer metastasis [84]. The CXCR4 receptor is first identified as a co-receptor that allows HIV to enter lymphocytes [85]. On the other hand, another receptor called CXCR7, which was renamed ACKR3 (atypical chemokine receptor 3) in 2014, has been shown to bind CXCL12 with about ten times greater affinity than CXCR4 [86]. This receptor can also bind to CXCL11. One distinctive feature is that the CXCL12-ACKR3 complex does not activate G proteins but signals through the β-arrestin pathway [87].

It has been revealed that elevated expression of CXCL12 is significantly correlated with high tumor stage, the presence of lymph node and distant metastases [88,89]. Wang et al. demonstrated that CXCL12, CXCR4, and CXCR7 are expressed both in primary CRC tissue and in lung metastases samples. In addition, authors observed that the expression levels of both CXCL12 and CXCR7 were elevated in metastatic samples compared to primary tumor tissues [90]. Moreover, high tumor expression of CXCL12 has been associated with poor survival outcomes in CRC patients. Studies have further indicated that elevated expression levels of both CXCL12 and CXCR4 correlate with unfavorable prognosis in lymph node-positive cases, as well as with tumor-stroma ratio (TSR) and lymphocytic infiltration at the invasive front (LIR) [91,92]. Researchers have indicated that CRC patients whose tumors exhibit strong co-expression of CXCR4 and VEGF (Vascular Endothelial Growth Factor) in more than 50% of cells have particularly poor prognoses, with this expression profile serving as a strong and independent predictor of early distant metastasis [93]. Notably, the upregulation of CXCR4 expression has also been observed under hypoxic conditions, mediated by the activity of hypoxia-inducible factor 1-alpha (HIF-1α) [94]. Yang et al. reported that positive CXCR7 expression correlates with the presence of lymph node involvement, distant metastasis, and more advanced tumor–node–metastasis (TNM) staging in CRC [95]. Similarly, Sherif et al. found the immunohistochemical expression of cytoplasmic CXCR7 in 11% of colorectal adenomas and in 72.4% of CRC cases with a significant difference between both (*p* < 0.001) [96].

Although numerous studies suggest that high expression of CXCR4 and CXCR7 is associated with poor prognosis in CRC, other findings indicate that the role of these receptors may vary depending on the biological and clinical context. Analyses of the gene expression profiles of *CXCL12* and its receptors *CXCR4* and *CXCR7* in a large CRC patient cohort (*n* = 107) revealed a significant down-regulation of *CXCL12* mRNA in tumor tissues compared to matched normal colorectal mucosa, indicating a potential tumor suppressor function of this chemokine. The selected genes were validated in tumor specimens by reverse transcription–quantitative polymerase chain reaction (RQ-PCR) using TaqMan^®^ assays (Applied Biosystems, Thermo Fisher Scientific, Waltham, MA, USA). Notably, this reduction was observed not only in invasive tumors but also in precursor lesions such as polyps. From a clinicopathological perspective, reduced mRNA expression levels of *CXCL12, CXCR4*, and *CXCR7* in tumor tissues were significantly associated with adverse tumor characteristics, including increased tumor size, local invasion, poor histological differentiation, advanced tumor stage, nodal involvement, and lymphovascular invasion. Furthermore, lower expressions of *CXCL12* and *CXCR7* were predominantly observed in tumors located in the proximal colon, an anatomical region that is frequently associated with microsatellite instability (MSI), which suggests a potential role of this chemokine axis in the molecular subtype of colorectal cancer [97].

A study by Stanisavljevic et al. demonstrated that expression of CXCL12 and relative of the CXCL12–CXCR4 axis are independent predictors of 5-year disease-free survival (DFS) in TNM stage III colon cancer [98]. Although only a few drugs targeting the CXCL12–CXCR4 axis have been approved for clinical use. Several CXCR4 antagonists are being explored in cancer therapies. These include AMD3100 (Plerixafor) (Cambridge, Massachusetts, USA) T22, and peptide analogs that block the interaction between CXCL12 and its receptors CXCR4 and CXCR7. Clinical trials with CXCR4 inhibitors, such as CTCE-9908 and LY2510924, have shown promising antitumor effects in CRC [99,100,101].

**Figure 2:**
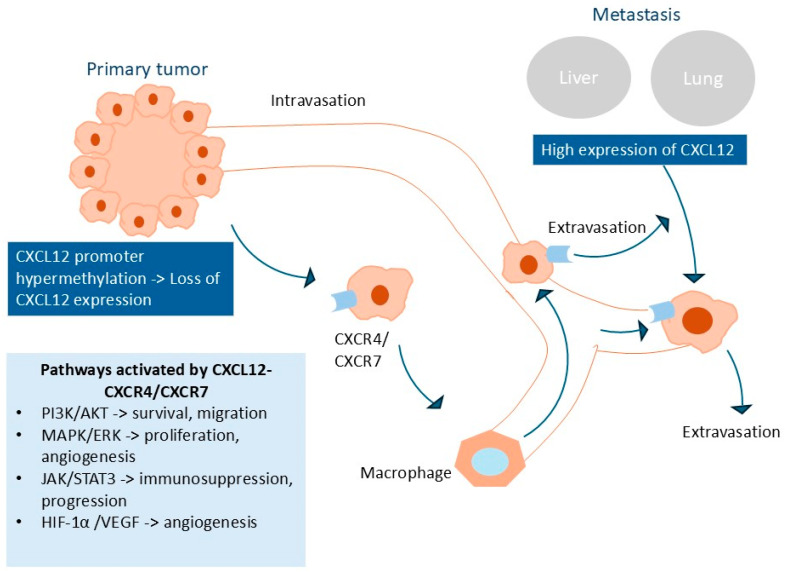
Mechanism of CXCL12-CXCR4/CXCR7 axis in colorectal cancer metastasis.

Clinical studies have shown that the CXCL12–CXCR4/CXCR7 axis plays a role in survival, tumor growth, angiogenesis, metastasis, the tumor microenvironment, and drug resistance, making it a promising target for cancer treatment. One hypothesis is that prior to the development of metastasis, many colon cancer cells (CRCs) undergo DNA hypermethylation within the CXCL12 promoter, leading to reduced autocrine and paracrine CXCL12 signaling [99]. As a result, CRCs can migrate along a gradient that leads them to distant organs known for high expression of this chemokine [45]. This process could be initiated early in colon carcinogenesis, as CXCL12 expression is lost already at the adenoma stage [57]. Beyond this, ligand binding activates multiple intracellular signaling pathways, including PI3K/AKT, MAPK/ERK, JAK/STAT3, and HIF-1α, which collectively promote tumor cell survival, proliferation, angiogenesis, and metastatic colonization. This process is shown in Figure 2.

#### 3.2.2. CXCL13, Its Receptors, and Related Signaling Pathways in Colorectal Cancer

CXCL13 is a chemotactic protein targeting B lymphocytes, which is mainly expressed by lymphoid organs, stromal cells. However, growing body of evidence suggests that this cytokine may also be secreted by some types of tumor cells [102,103,104,105]. It was proved that CXCL13 promotes the synthesis of various cytokines including IL-12 and IL-17 as well as activates the Wnt/β-catenin pathway [106,107,108]. In addition, receptor for CXCL13—CXCR5 is expressed in specific T lymphocyte subsets [109]. It was reported that CXCL13 binds to CXCR5, leading to lymphocytes migration and promotion of inflammation [110]. Furthermore, evidence suggests that CXCL13, together with CXCR5, may play a crucial role in regulating tumor occurrence and metastasis via the PI3K/AKT pathway, as demonstrated in studies on CRCs [110,111,112,113]. Emerging observations reported that CXCL13 can modulate tumor progression, such as the tumor-specific immune response, angiogenesis and metastasis [114,115]. The study of Luo et al. [105] has investigated that CXCL13 were sufficiently expressed in CRC tissues and positively correlated with clinicopathological features such as more advanced TNM stage, a greater depth of tumor invasion (T stage) and the presence of lymph node metastases (N stage) [105]. Similar results were obtained by Qi et al. [115], who also showed that the expression of CXCL13 and its receptor CXCR5 were significantly increased in CRC tissues compared with adjacent cancer cells, which was more apparent in tumors with higher tumor staging (T3 stage). CXCL13 expression was also associated with poor OS rate, what was confirmed by other authors [102,106]. The findings presented demonstrated that CXCL13 might be a potential indicator for tumor features and survival in patients with CRC [106].

A growing body of evidence has demonstrated the important role of CXCL13 in promoting resistance to 5-fluorouracil (5-Fu) in CRC [102]. This resistance is associated with abnormal activation of the PI3K–Akt signaling pathway, deregulated expression of cell cycle–related proteins, dysregulation of specific miRNAs, and promoter hypermethylation of resistance-related genes [102,115,116]. In the study by Zhang et al., the authors evaluated the role of CXCL13 in 5-Fu resistance using both cell-based experiments and clinical samples [102]. Functional assays, including cytokine arrays and culture medium exchange experiments, revealed that CXCL13 protein levels were significantly elevated in the supernatants of 5-Fu–resistant CRC lines compared to non-resistant lines. These findings indicate increased CXCL13 secretion by resistant cells, rather than changes in intracellular expression alone. To assess clinical relevance, serum concentrations of CXCL13 were measured using ELISA in patients stratified by 5-Fu treatment response. The results showed that patients with 5-Fu–resistant CRC had significantly higher serum levels of CXCL13 compared to 5-Fu–sensitive individuals [102]. Furthermore, elevated CXCL13 levels in serum were associated with poorer clinical outcomes, including reduced OS and DFS [102]. Qi et al. [114] reported the significant differences in elevated expression of CXCL13 and more advanced TNM stage, distant metastasis, differentiation and neural invasion, while CXCR5 and CXCL13 overexpression was associated with poor correlation with the OS and RFS [116]. The role of CXCL13 as a potential importance in CRC therapy has been confirmed by other investigators, who also revealed that CXCL13/CXCR5 axis may target tumors by recruiting B lymphocytes, and is essential in the anti-tumor immune response of TME in CRC [109,116]. Authors confirm a novel target for 5-Fu–resistant CRC in prevention and treatment strategies in this malignancy [102].

#### 3.2.3. CXCL14, Its Receptors, and Related Signaling Pathways in Colorectal Cancer

CXCL14 is a novel member of the chemokine family and plays important role in maintaining a self-stable physiological function [117]. CXCL14 as a homeostasis-associated chemokine, may promote the development of CRC by blocking the chemotaxis of immune cells. However, the growing body of evidence revealed that the role of CXCL14 in cancer is still not clear [118]. Lin et al. [117], who evaluated CXCL14 expression in colorectal carcinoma tissues and adjacent normal mucosa using RT-qPCR (mRNA level) and IHC (protein level), reported that both CXCL14 mRNA and protein were significantly reduced in CRC tissues compared to normal controls (*p* < 0.05). Zeng et al. demonstrated that the expression of CXCL14 was found to be elevated in CRC tissue in comparison to normal colon tissue [119]. The conflicting results regarding CXCL14 expression in CRC may be due to differences in the definition of control tissue, assessment methods (RT-qPCR vs. IHC scoring), tumor heterogeneity, and the dual biological role of CXCL14 (tumor suppressor vs. tumor promoter). Clinical correlation analysis indicated that low CXCL14 expression in tumor tissues was significantly associated with presence of lymph node metastasis, tumor location, and advanced clinicopathological stage (*p* < 0.05). Furthermore, Kaplan–Meier survival analysis and Cox regression revealed that reduced CXCL14 expression correlated with poor prognosis (*p* < 0.01). To investigate the functional role of CXCL14 at the cellular level, a replication-defective lentiviral vector overexpressing CXCL14 was constructed and transfected into HT29 colorectal cancer cells. Cell proliferation was assessed using the Cell Counting Kit-8 assay (Dojindo Molecular Technologies, Kumamoto, Japan) and cell cycle distribution was evaluated by flow cytometry. Overexpression of CXCL14 inhibited cell proliferation and induced G1-phase cell cycle arrest. Other clinical investigations suggest that the reduced CXCL14 expression in CRC may result in local immune deficiencies such as weakened immune surveillance and presentation of antigens, immune evasion, as well as disordered internal immune environment, what may cause by low permeability of immune cells in tissues [118]. Cao et al. revealed that promoter hypermethylation of the CXCL14 gene, detected by methylation-specific PCR, was observed in ~79% of primary CRC samples and led to silencing of CXCL14 expression in CRCs. Restoration of CXCL14 expressions was found to inhibit CRC proliferation. Additionally, CXCL14 suppressed migration, invasion, and EMT by downregulating NF-κB signaling [120].

#### 3.2.4. CXCL16, Its Receptor, and Related Signaling Pathways in Colorectal Cancer

CXCL16 exists in two biologically active forms: a membrane-bound variant (mCXCL16) and a soluble form (sCXCL16), generated through proteolytic cleavage [121,122]. The two forms of CXCL16 serve distinct biological roles. The sCXCL16 functions as a chemokine, attracting CXCR6-expressing cells through chemotaxis. In contrast, mCXCL16 is a transmembrane protein that may act as an adhesion molecule by interacting with C-X-C chemokine receptor 6 (CXCR6) [121]. Notably, this adhesion does not require signal transduction through the CXCR6 receptor. This function of mCXCL16 is particularly significant during inflammation, where pro-inflammatory cytokines upregulate its expression on vascular endothelium, promoting immune cell recruitment to the affected tissue [121,122]. Chen et al. demonstrated that CXCL16 expression was significantly elevated in CRC tissue and was negatively correlated with OS in patients. Subgroup analysis showed that patients with positive CXCL16 expression in TNM stage III/IV had significantly lower OS rates compared with those with negative expressions. In contrast, no significant difference was observed in TNM stage I/II, which the authors attributed to the small sample size in this subgroup. In multivariate Cox regression analysis, positive CXCL16 expression was identified as an independent prognostic factor associated with poorer OS in CRC patients [122]. CXCL16 expression in the TME has been shown to be closely associated with the presence oPfBRAF mutations. In the study conducted by Deng et at. observed that CXCL16 could enhance tumor metastasis by influencing angiogenesis within the TME of BRAF V600E-mutant CRC [122]. Elevated CXCL16 expression were linked to unfavorable prognosis through the promotion of angiogenesis in the TME. These insights are essential for improving outcomes in patients with BRAF V600E mutations and may help inform anti-angiogenic treatment strategies [122].

CXCR6, the receptor for the CXCL16, is expressed on memory T cells and activated Th1 and Tc1 effector T cells. This receptor helps guide these cells to sites of inflammation, highlighting its role in immune responses and its potential relevance in cancer immunotherapy [123]. Wang et al. conducted a comprehensive study on the role of CXCR6 in CRC, showing that its expression was higher in tumor tissues than in the surrounding normal tissues and that this higher expression correlated with better patient prognosis. They emphasized that CXCR6 plays an important role in boosting the effectiveness of programmed cell death protein 1 (anti-PD-1) therapy and suggested that the elevated expression of this receptor is driven by factors within the tumor tissue itself rather than by chemotactic signal [124]. In the study by Liu et al., it was demonstrated that silencing CXCR6 enhances the migration and invasion of CRCs [125]. Transcriptome analysis revealed a link between CXCR6 and the activation of the Vascular Endothelial Growth Factor A/Phosphoinositide 3-Kinase/Protein Kinase B/Mechanistic Target of Rapamyci (VEGFA/PI3K/AKT/mTOR) signaling pathway. Importantly, inhibition of either VEGFA or the PI3K/AKT/mTOR pathway reversed the increased migratory and invasive abilities of CRCs following CXCR6 silencing, highlighting the receptor’s key role in regulating tumor aggressiveness [125].

### 3.3. Cross-Talk and Subtype-Specific Roles of CXC Chemokines

CXC chemokines in CRC operate within overlapping networks that exhibit considerable pathway cross-talk. A number of ligands, including CXCL1, CXCL2, CXCL5, and CXCL8, converge on CXCR2, thereby coordinating neutrophil recruitment, angiogenesis, epithelial–mesenchymal transition, and tumor proliferation. Through additional mechanisms, CXCL2 can activate Gαi-2/Gαq-11 signaling to promote macrophage recruitment and resistance to irinotecan, while CXCL5, regulated by Basic Leucine Zipper ATF-Like Transcription Factor 3 (BATF3), enhances angiogenesis and prognosis prediction. In contrast, the CXCL12/CXCR4 axis, widely implicated across multiple cancer types, drives PI3K/AKT and PI3K/AKT/mTOR signaling, macrophage recruitment, and metastatic spread, particularly to the liver [126]. Importantly, emerging evidence suggests that chemokine activity may be influenced by molecular subtypes of CRC. Bogomolova et al. reported that the CXCL8–CXCR1 axis is upregulated in BRAF-mutant tumors, with significantly higher CXCR1 expression (*p* = 0.009). Elevated CXCR1 correlated with poorer tumor differentiation and reduced sensitivity to standard chemotherapy regimens (FOLFOX/XELOX), indicating that chemokine pathways may contribute to therapy resistance in this subgroup [127]. Consensus Molecular Subtype 1 (CMS1), one of the molecular subtypes of CRC, is strongly associated with MSI-high status. These tumors are characterized by increased expression of T- and B-cell–attracting chemokines such as CXCL9, CXCL10, CXCL16, and CXCL13, consistent with their prominent lymphocytic infiltration and improved prognosis. Conversely, the CMS4 mesenchymal subtype shows enrichment in CXCL12 and CCL2, promoting immunosuppressive cell recruitment and angiogenesis [128]. Table 2 shows a role of discussed chemokines and their receptors in the pathogenesis of CRC and their biomarker potential.

## 4. Advanced Technologies and Clinical Perspectives in Colorectal Cancer

Recent research highlights several promising clinical avenues in CRC. Surface-enhanced Raman spectroscopy (SERS) enables sensitive, multiplexed phenotyping of tumor cell surface proteins and shows potential as a minimally invasive diagnostic tool for monitoring treatment response. In particular, by distinguishing KRAS mutant from wild-type phenotypes under anti-EGFR therapy, SERS could support more precise patient stratification and guide targeted therapeutic decisions [129]. Chemoresistance to oxaliplatin remains a major clinical challenge [130]. Insights from multi-omics analyses of resistant cells indicate that chromatin structural remodeling contributes to enhanced metastatic potential and altered redox balance. These findings open perspectives for the development of novel biomarkers to identify resistant tumors early and for targeting 3D genome architecture or associated pathways to restore chemosensitivity. The discovery that Fusobacterium nucleatum colonizes CRC tissues and promotes tumor progression, immune suppression, and therapy resistance highlights an underexplored therapeutic target [131]. Clinical strategies beyond conventional antibiotics including natural products, engineered polymers, bacteriophages, probiotics, and vaccines—may provide innovative ways to disrupt tumor-associated microbiota and improve treatment outcomes. Finally, the integration of ferroptosis induction with immunotherapy offers an emerging treatment paradigm [132]. Nanoplatform-based systems that combine ferroptosis triggers with immune activation not only enhance tumor immunogenicity but also generate a self-sustaining cancer–immunity cycle. This dual approach has shown efficacy in both primary and metastatic CRC models and may pave the way for synergistic therapies that overcome resistance to conventional treatments.

## 5. Conclusions

CRC remains one of the most serious oncological challenges in personalized medicine. An important goal for the future is to develop innovative diagnostic strategies centered on novel and precise tumor biomarkers, aimed at enhancing the early detection of cancers such as CRC. Chemokines play a critical role in the regulation of homeostasis under normal physiological conditions and in pathological processes. This review highlights the potential of selected ELR+ and ELR- chemokines and their receptors as promising candidates for biomarkers and therapeutic targets in CRC. CXCL1 showed elevated expression in CRC tissues that correlated with more advanced clinicopathological features, such as larger tumor size, deeper invasion, the presence of lymph node metastasis, and higher clinical stage, thus is being considered a potential biomarker of tumor progression. Similarly, expression of CXCL8 in tumor tissues and/or serum concentrations of this chemokine were significantly elevated in CRC patients and associated with more advanced stages of tumor, the presence of distant metastases and poorer clinical outcomes, supporting its role as a potential prognostic biomarker and therapeutic target. The observed discrepancies regarding the CXCL12–CXCR4/CXCR7 axis largely reflect differences in analytical methods. Studies using IHC have reported increased protein expression in CRC tissue, correlating with advanced stage, metastasis, and poor survival, whereas large-scale RT-qPCR analyses demonstrated downregulation of CXCL12, CXCR4, and CXCR7 mRNA compared with normal mucosa. These contrasting results, further influenced by cohort heterogeneity and tumor location, emphasize the need for standardized methodologies and careful patient stratification to determine the true clinical value of these molecules as biomarkers and therapeutic targets. Elevated expression of CXCL13 was correlated with poorer prognosis, nodal involvement, and more advanced stage of CRC. The CXCL13/CXCR5 axis was reported to be involved in regulating cancer cell migration and shaping an immunosuppressive TME, highlighting its potential as a prognostic biomarker and therapeutic target. Moreover, CXCL14 showed reduced expression in CRC tissues compared to normal tissue, with significantly lower levels observed in advanced stages of the disease. On contrary, some studies have reported increased expression of this chemokine in CRC when compared to normal tissue. These discrepancies may be related to methodological differences, control tissue selection and tumor heterogeneity. Individuals with positive CXCL16 expression in advanced stages of CRC exhibit reduced OS compared to those lacking expression of this chemokine. Therefore, CXCL16 may be considered an independent prognostic factor for OS in CRC.

In summary, chemokines and their receptors are important contributors to the development and progression of CRC, affecting tumor growth, immune regulation, and metastasis. Their altered levels in tumor tissues and/or blood of CRC patients were correlated to clinical outcomes, which makes them promising candidates for assessment of progression and early diagnosis of CRC. Some chemokines also show potential as therapeutic targets. Further studies, especially those evaluating serum levels, are necessary to confirm their usefulness in clinical practice.

## Figures and Tables

**Table 2:** Role of Selected Chemokines and Its Receptor in the Pathogenesis of CRC and Their Biomarker Potential.

Chemokine	Receptor(s)	Role in CRC	Expression: Tissues/Serum	Biomarker Potential	References
CXCL8	CXCR1 and CXCR2	Promotes proliferation, invasion, angiogenesis, immune evasion	Elevated serum levels in CRC patients and overexpressed in CRC tissue	Diagnostic and prognostic marker—elevated tissue and serum levels correlate with advanced tumor stage, presence of distant metastases, and shorter OS and RFS.	[56,57,58,59,60,61,62,63,64,65,66,67,68,69,70]
CXCL1	CXCR2	Promotes proliferation, migration, angiogenesis	Overexpressed in CRC tissue	Prognostic marker—associated with poor survival, larger tumor size, deeper invasion, lymph node metastasis, and advanced clinical stage.	[71,72,73,74,75,76,77,78,79,80,81,82]
CXCL12	CXCR4 and CXCR7	Enhances metastasis, chemoresistance	Overexpressed in CRC tissue	Prognostic marker—tissue expression linked to lymphatic and distant metastasis and reduced DFS.	[83,84,85,86,87,88,89,90,91,92,93,94,95,96,97,98,99,100,101]
CXCL13	CXCR5	Promotes immune cell recruitment, tumor progression	Elevated serum levels in CRC patients and overexpressed in CRC tissue	Prognostic marker—associated with advanced TNM stage, lymph node involvement, and poor OS and DFS; elevated serum levels also predict 5-Fu resistance and worse clinical outcome.	[102,103,104,105,106,107,108,109,110,111,112,113,114,115,116]
CXCL14	Unknown	Promotes or suppresses tumor progression	Overexpressed in CRC tissue	Prognostic marker—reduced expression in CRC tissues associated with advanced stage and decreased OS; may function as a tumor suppressor depending on cellular source.	[117,118,119,120]
CXCL16	CXCR6	Promotes migration, invasion, immune modulation	Overexpressed in CRC tissue	High expression linked to poor OS in stage III/IV CRC; associated with angiogenesis and unfavorable outcomes in BRAF-mutant tumors.	[121,122,123,124,125]
